# Specific Link between Lung and Large Intestine: A New Perspective on Neuropeptide Secretion in Lung with Herbal Laxative Stimulation

**DOI:** 10.1155/2013/547837

**Published:** 2013-08-20

**Authors:** Xiang-Gen Zhong, Feng-Jie Zheng, Yu-Hang Li, Hong Xu, Qian Wang, Yu-Chao Liu, Miao Liu, Ruo-Han Wu, Yu-Shan Gao, Shu-Jing Zhang, Jin-Chao Zhang, Tian-Yu Zhang, Si-Hua Gao

**Affiliations:** School of Preclinical Medicine, Beijing University of Chinese Medicine, Beijing 100029, China

## Abstract

*Background*. To investigate the specific link between lung and large intestine. *Methods*. Rat COPD-like model was prepared. Mirabilite or Chinese rhubarb was administrated intragastrically to stimulate the large intestine. Histological analysis of lung inflammation was assessed. The tissues levels of SP, VIP, NK1R, VIPR1, and VIPR2 were measured by using ELISA kits. In addition, mouse model of allergic asthma was prepared. Mirabilite was administrated intragastrically to stimulate the large intestine. Airway responsiveness and lung inflammation were assessed. The tissues levels of SP, VIP, NKA, NKB, NK1R, VIPR1, and VIPR2 were measured by using ELISA kits. *Results*. Stimulating the intestine with Mangxiao or Dahuang, SP, NK-1R, VIP, VIPR1, and VIPR2 were significantly increased in intestine tissues of rats with COPD and mice with asthma. Meanwhile, the SP and NK1R were significantly decreased, while VIP, VIPR1, and VIPR2 were significantly increased in lung tissues. An abnormal secretion of SP and VIP can be observed in other tissues; however, no marked changes were found in the receptors. The NKA and NKB levels were similar in lung tissues of mice with asthma among groups. *Conclusions*. Stimulating intestine with Mangxiao or Dahuang can specifically regulate the secretion of SP, VIP, and the receptors in lung tissues.

## 1. Introduction

Traditional Chinese medicine (TCM) is characterized by its unique system of therapies and theories. The theory of exterior-interior correlation between the lung and large intestine is one of the most important components of the TCM Zang-Fu theory, which has been widely applied in TCM clinical practice, particularly in the treatments of the lung-intestine diseases, such as COPD and asthma [1]. Over thousands of years, TCM doctors have been continuously exploring the practical implication and application of the theory. They found that the significance of the theory is demonstrated in the explanation of lung and large intestine physiological functions.

Recent studies have proved the relationship between lung and large intestine from various perspectives, such as embryonic development, lymph circulation, mucosal immunity, micro ecology, and inflammatory harass [2, 3]. However, this exterior-interior relationship between lung and large intestine still has not been completely explicated. Further research on this theory from a biological point of view is necessary, which we believe might provide new valuable references for the treatment strategies for lung-intestine diseases.

Relaxing the bowels with laxative is one of the common treating methods for lung diseases based on the theory of exterior-interior correlation between lung and large intestine. Our previous studies found that Chinese Rhubarb (Dahuang) could not only effectively improve the intestinal obstruction in COPD patients and rats with COPD but also effectively improve the dyspnea and gas exchange function [4, 5], and this effect was related to the changes of neuropeptide levels in the blood [6]. Therefore, we hypothesized that stimulus of intestinal track, like herbal laxatives (osmotic or stimulant), may affect the secretion of certain specific neuropeptides (factors linked with lung performance) in lung tissue, which is similar to the inner meridians collaterals between lung and large intestine. In order to confirm this hypothesis, we conducted the following study.

## 2. Materials and Methods

### 2.1. Preparation of Herbs

Mangxiao and Dahuang were purchased from a traditional Chinese medicinal store (Tongrentang) in Beijing, China, and were authenticated by Professor Yue-qi Wang, Basic Theory and Key Technology Research Center, Beijing University of Chinese Medicine. Mangxiao is an osmotic laxative, acting on large intestine, and its effective ingredient is sodium sulfate [7]. Dahuang is a stimulant laxative, acting on large intestine, and its main effective ingredient is phenolphthalein [7]. According to the dose-equivalence equation between rats and humans (9 g/d) [8], the dose of Mangxiao and Dahuang for rats was 1.5 g/kg, and the dose of Mangxiao for mice was 1.0 g/kg. Mangxiao or Dahuang decoction was prepared in accordance with conventional TCM decocting methods, respectively [9, 10]. Quality was controlled with high-performance liquid chromatography (HPLC) and ion chromatography [11, 12]. The herbs were nontoxic, which were evaluated by animals liver and kidney function, and so on (data not shown).

### 2.2. Experimental Animals

Male Wistar rats (230 ± 20 g) and female BALB/c mice (20 ± 2 g) were obtained from Bei Jing Vital River Laboratories (VRL) Co., Ltd. (Beijing, China), and maintained in the Animal Center, Beijing University of Chinese Medicine, for 1 week prior to the experiments. Animals were maintained in plastic cages at 23°C ± 1°C with free access to water. They were kept on a 12-hour light-dark cycle. All experimental procedures were performed in accordance with the guidelines of the Animal Care and Ethics Committee of Beijing University of Chinese Medicine.

### 2.3. Rat COPD-Like Model and Herb Administration

Rat COPD-like model was prepared by cigarette smoking and lipopolysaccharides (LPS, Sigma, St. Louis, MO, USA) stimuli, as previously described [13]. Briefly, on the 1st and 14th days, 200 *μ*L LPS (1 mg/mL in normal saline) was administered intratracheally. On days 2–28, rats were confined in an airtight plexiglass container (60 cm × 50 cm × 40 cm) for 1 hour twice daily, which was full of cigarette smoke at a concentration of about 5% (v/v). Rats were randomized into 4 groups, the treatment was performed as follows: control group received room air, intratracheal normal saline instillation, and intragastric administration of water on days 22–28; model group received cigarette smoking, intratracheal LPS instillation, and intragastric administration of water on days 22–28; Mangxiao group received cigarette smoking, intratracheal LPS instillation, and intragastric administration of Mangxiao decoction on days 22–28; Dahuang group received cigarette smoking, intratracheal LPS instillation, and intragastric administration of Dahuang decoction on days 22–28.

### 2.4. Mouse Model of Allergic Asthma and Herb Administration

Sensitization and challenge of mice were performed as previously described [14], with some modifications. Briefly, mice were immunized via intraperitoneal injection of 100 *μ*g chicken OVA (grade V; Sigma, St. Louis, MO, USA) and 4 mg aluminum hydroxide (Thermo, Rockford, IL, USA) suspended in 0.1 mL saline on days 0 and 14. On days 22, 23, and 24, mice were challenged with aerosolized 3% (w/v in PBS) OVA solution for 30 min using an ultrasonic nebulizer (YC-Y800, Yadu Corp., Beijing, China). Mice were randomly divided into 3 groups, the treatment was performed as follows: control group and model group received intragastric administration of water on days 17–23; Mangxiao group received intragastric administration of Mangxiao decoction on days 17–23. Animals were sacrificed 48 h after the last challenge.

### 2.5. Assessment of Respiratory Resistance in Mouse Model of Allergic Asthma

Airway high reactivity (AHR) as an indicator of decline in lung function was detected in 24 hrs after the final exposure to aerosol by using an AniRes 2005 Lung Function system (Bestlab 2.0, Beijing, China) according to manufacturer's instructions. Anesthesia was induced by intraperitoneal injection of 95 mg/kg pentobarbital sodium. A connection was made by a computer-controlled ventilator *via* a cannula that had been implanted surgically in the trachea. The respiratory rate and the time ratio of expiration/inspiration were preset at 90/min and 1.5 : 1, respectively. Each mouse inhaled increasing doses of methacholine (MCH, Sigma, USA) ranging from 0 to 0.2 mg/kg body weight for 5 min while staying inside the whole-body plethysmograph, and AHR was recorded and assessed by indexes of expiratory resistance (*R*_*e*_).

### 2.6. Histological Analysis of Lung Inflammation in Rat Model of COPD and Mouse Model of Allergic Asthma

For staining with H&E, lungs were inflated and fixed with 10% buffered formalin. Samples were embedded in paraffin, then sectioned (4 *μ*m), and stained with H&E.

### 2.7. Measurement of SP and VIP in the Lung, Large Intestine, Stomach, Kidney, Spleen, Heart, Brain, and Liver Tissues in Model of COPD and Allergic Asthma

After the serum sample collection, lung, large intestine, stomach, kidney, spleen, heart, brain, and liver tissues were rinsed in ice cold phosphate-buffered saline (PBS: pH 7.5), then dried, and weighed. Afterwards, the tissues were homogenized by using a glass homogenizer on ice, with 10 mL/g of ice cold PBS. Next, homogenates were centrifuged at 5000 ×g, for 5 min at 4°C, and supernatants were collected for detection. The tissue levels of SP and VIP were measured by using enzyme-linked immuno sorbent assay (ELISA) kits according to manufacturer's instructions (Cusabio). 

### 2.8. Measurement of NK1R, VIPR1, and VIPR2 in Lung, Large Intestine, Stomach, And So On Tissues in Model of COPD and Allergic Asthma

After the serum sample collection, lung, large intestine, stomach, heart, and brain tissues were rinsed in ice cold phosphate-buffered saline. Then, the rinsed tissues were homogenized in 1 mL PBS and stored overnight at −20°C. After two freeze-thaw cycles, the tissues were performed to break the cell membranes, the homogenates were centrifuged at 5000 ×g, for 5 minutes at 4°C, and the supernatants were collected for detection. 

Levels of NK1R in the tissues of lung, large intestine, stomach, heart, and brain, VIPR1 and VIPR2 levels in the tissues of lung, large intestine, stomach, and brain of COPD rats, and levels of NK1R, VIPR1, and VIPR2 in the tissues of lung, large intestine, stomach, and heart of allergic asthma mice were measured by using a commercial ELISA kit, following the manufacturer's instructions (Cusabio).

### 2.9. Measurement of NKA and NKB in Lung, Large Intestine, Stomach, Kidney, Spleen, Heart, Brain, and Liver Tissues in Model of Allergic Asthma

Levels of NKA and NKB in lung, large intestine, stomach, kidney, spleen, heart, brain, and liver tissues were measured by using a commercial ELISA kit, following the manufacturer's instructions (Cusabio).

### 2.10. Statistical Analysis

Data were expressed as mean values ± standard deviation. Statistical comparisons were performed by using one-way analysis of variance. Significant levels were set at *P* < 0.05.

## 3. Results

### 3.1. Effects of Mangxiao or Dahuang on SP and VIP Levels in Lung, Large Intestine, Stomach, Kidney, Spleen, Heart, Brain, and Liver Tissues of COPD-Like Rats

Compared with the control group, the levels of SP in lung, stomach, and brain tissues were significantly increased, while the levels of SP in large intestine and heart tissues were decreased in the model group (*P* < 0.05). Compared with the model group, the levels of SP in lung, stomach, and brain tissues were significantly decreased, while the level of SP in large intestine tissue were increased in Mangxiao group and Dahuang group (Table 1).

The levels of VIP in lung, large intestine, and brain tissues in the model group were notably lower than that in the control group (*P* < 0.05). However, intervention with Mangxiao or Dahuang significantly increased the levels of VIP in lung and large intestine tissues while reducing the levels of VIP in brain tissue compared with the model group (*P* < 0.05) (Table 2).

### 3.2. Effects of Mangxiao or Dahuang on NK1R, VIPR1, and VIPR2 Levels in Lung, Large Intestine, Stomach Heart, and Brain Tissues of COPD-Like Rats

Compared with the control group, the level of NK1R in lung tissues was distinctively increased, while the levels of NK1R in large intestine and brain tissues were decreased in the model group. Intervention with Mangxiao or Dahuang significantly reduced the level of NK1R in lung tissue (*P* < 0.05) compared with the model group (Table 3).

The levels of VIPR1 and VIPR2 in lung and large intestine tissues in the model group were significantly lower than that in the control group. Intervention with Mangxiao or Dahuang increased the levels of VIPR1 and VIPR2 in lung tissues, compared with the model group, while the levels of VIPR1 and VIPR2 in stomach and brain tissues had no obvious changes (Tables 4 and 5).

### 3.3. Effects of Mangxiao on SP and VIP Levels in Lung, Large Intestine, Stomach, Kidney, Spleen, Heart, Brain, and Liver Tissues of OVA Allergic Asthma Mice

Compared with the control group, a considerable increase in the level of SP in lung tissues and a decrease in the levels in large intestine, heart, and liver tissues were observed in the model group. Intervention with Mangxiao markedly lowered the level of SP in lung tissues and increased the levels of SP in large intestine and stomach tissues (Table 6).

Compared with the control group, the levels of VIP in lung, large intestine, heart, and brain tissues were reduced significantly (*P* < 0.05), and the level of VIP in stomach tissues was increased obviously in the model group. Intervention with Mangxiao increased the level of VIP in lung, large intestine, and heart tissues while lowering the level of VIP in stomach tissues compared with that in the model group (Table 7).

### 3.4. Effects of Mangxiao on of NK1R, VIPR1, and VIPR2 Levels in Lung, Large Intestine, Stomach, and Heart Tissues of OVA Allergic Asthma Mice

Compared with the control group, the level of NK1R in lung and stomach tissues was up (*P* < 0.05), while the levels of NK1R in large intestine and heart tissues had no distinct changes in the model group mice. Compared with the model group, the level of NK1R in lung tissues reduced obviously, while the level of NK1R in large intestine tissues increased sharply in the Mangxiao group (Table 8).

Compared with the control group, the levels of VIPR1 and VIPR2 in lung tissue were significantly lower (*P* < 0.05). Intervention with Mangxiao visibly increased (*P* < 0.05) the level of VIPR1 in lung tissues compared with the model group (Tables 9 and 10). 

### 3.5. Effects of Mangxiao on NKA and NKB Levels in Lung, Large Intestine, Stomach, Kidney, Spleen, Heart, Brain, and Liver Tissues of OVA Allergic Asthma Mice

The levels of NKA and NKB in lung, large intestine, stomach, kidney, and spleen tissues were similar between the control group and the model group (*P* > 0.05). Compared with the control group, levels of NKA in heart and liver tissues and level of NKB in brain tissues went down significantly (*P* < 0.05). However, the levels of NKA in large intestine and NKB levels in lung, large intestine, stomach, and spleen tissues were reduced in Mangxiao group (Tables 11 and 12). 

### 3.6. Effect of Mangxiao or Dahuang on Inflammation in Lung Tissues of COPD-Like Rats

**Figure 1:**
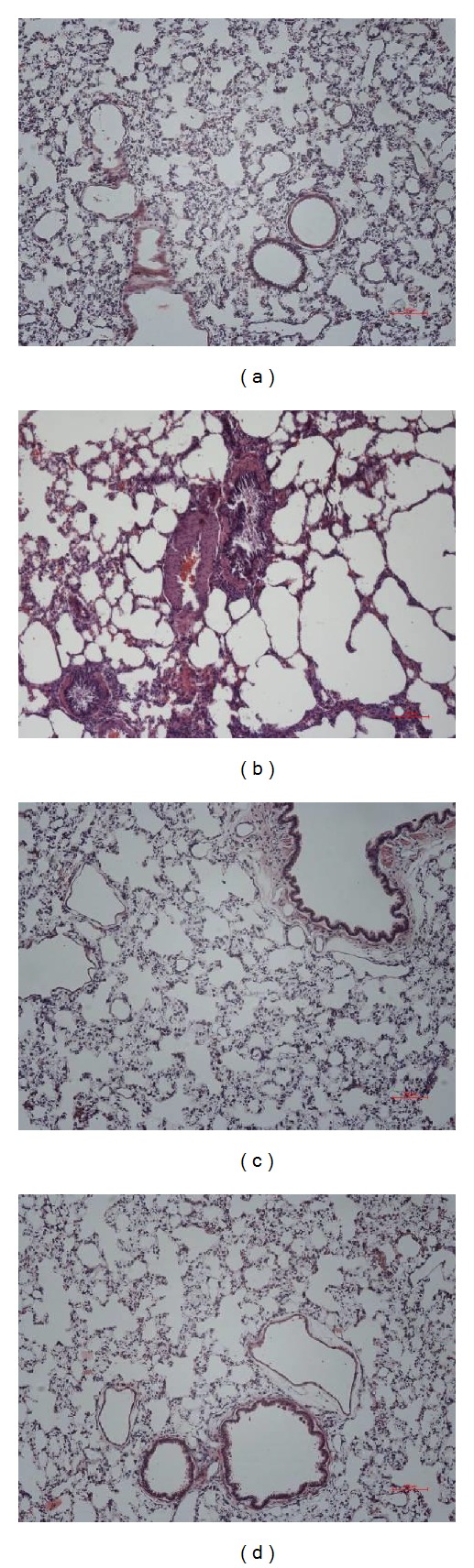
The effects of Mangxiao or Dahuang on COPD-like rats' lung histopathology (H&E stain ×100). Histological examination of lung tissues was performed 48 h after the final administration. Lung tissues were fixed, sectioned at 4 *μ*m thickness, and stained with H&E solution. (a) Control group: received room air, intratracheal normal saline instillation, and intragastric administration of water. (b) Model group (LPS + cigarette): received cigarette smoking, intratracheal LPS instillation, and intragastric administration of water. (c) Mangxiao group (LPS + cigarette + Mangxiao): received cigarette smoking, intratracheal LPS instillation, and intragastric administration of Mangxiao (1.5 g/kg) decoction. (d) Dahuang group (LPS + cigarette + Dahuang): received cigarette smoking, intratracheal LPS instillation, and intragastric administration of Dahuang (1.5 g/kg) decoction.

As the results of hematoxylin-eosin staining shown in Figure 1, the lung tissue of control group had normal alveolar structure and no inflammatory infiltration and exudates. The model group showed basic pulmonary pathological changes of COPD, the bronchial epithelium presented with degeneration, necrosis, and shedding, and intrabronchial presented with exudation. The bronchial wall was thickening and surrounded by pulmonary bullae. Compared with the model group, after treatment of Mangxiao or Dahuang, the pathological changes of lung tissue were alleviated, which presented with mild congestion and pulmonary interstitial inflammation (Figure 1).

### 3.7. Comparison of Airway Effect in Mice

**Figure 2:**
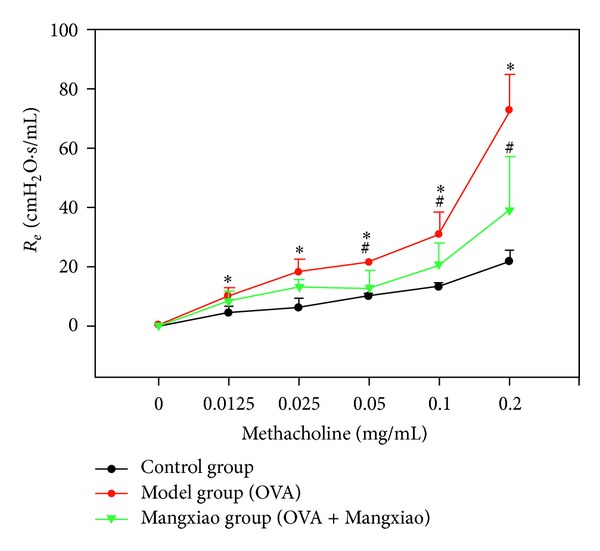
The development of airway hyperresponsiveness analysis (mean ± SD, *n* = 5). All values are expressed as mean ± SD. Means labeled with superscripts were significantly different. *P < 0.05 versus control group, ^#^ *P* < 0.05 versus model group (OVA).

After excitation by different concentrated MCH, airway resistance changes are displayed in Figure 2. Compared with the control group, when the MCH concentration was 0.0125 mg/kg, 0.025 mg/kg, 0.05 mg/kg, 0.1 mg/kg, and 0.2 mg/kg, respectively, the airway resistance in the model group mice went up distinctively (10.03 ± 2.63 versus 5.06 ± 2.38, 18.05 ± 4.38 versus 8.09 ± 4.52, 21.21 ± 0.57 versus 10.91 ± 1.98, 30.66 ± 7.67 versus 13.47 ± 1.52, and 104.15 ± 35.23 versus 22.66 ± 3.81); compared with the model group, when the MCH concentration was 0.05 mg/kg, 0.1 mg/kg, and 0.2 mg/kg, respectively, airway resistance decreased significantly in mice of Mangxiao group (12.55 ± 5.97, 20.39 ± 7.53, and 38.84 ± 18.28) (Figure 2).

### 3.8. Effect of Mangxiao on Inflammation in Lung Tissue of OVA Allergic Asthma Mice

**Figure 3:**
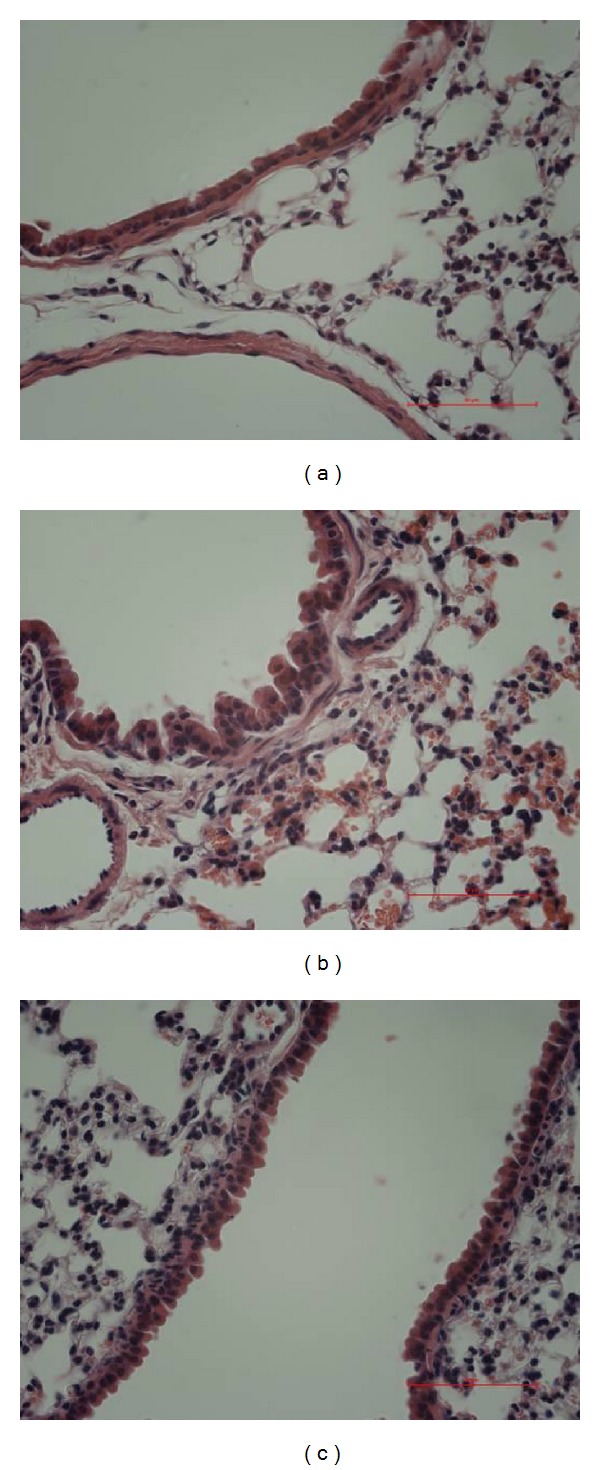
The effects of Mangxiao on mice's lung histopathology (H&E stain ×400). Histological examination of lung tissues was performed 48 h after the final OVA challenge. Lung tissues were fixed, sectioned at 4 *μ*m thickness, and stained with H&E solution. (a) Control group, (b) model group (OVA): OVA-sensitized/-challenged mice, and (c) Mangxiao group (OVA + Mangxiao): Mangxiao (1 g/kg) + OVA-sensitized/-challenged mice.

Hematoxylin-eosin staining displayed that alveolar epithelial cells arranged orderly, alveolar walls had structure integrity, no hyperemia, hemorrhage, or inflammatory cell infiltration were present in the control group. However, OVA-challenged lung tissues displayed bronchial epithelial cells which arranged disorderly. Compared to the control group, part of epithelium damage and infiltration of inflammatory cells into the airway were observed around the bronchi, bronchioles, and alveoli. Moreover, the majority of leukocytes were eosinophils and lymphocytes. Infiltration of inflammatory leukocytes in OVA-challenged mice treated with Mangxiao was significantly attenuated, compared with that in OVA-challenged mice (Figure 3).

## 4. Discussion

This study explored the specific link between lung and large intestine. Rat COPD-like model was prepared by cigarette smoking and LPS stimuli. Intragastric administration of Mangxiao (an osmotic laxative) or Dahuang (a stimulant laxative) was to stimulate the large intestine. Mouse model of allergic asthma was prepared by ovalbumin (OVA) sensitization and challenge. Intragastric administration of Mirabilite was to stimulate the large intestine. The tissue (including lung, and large intestine) levels of neuropeptides and the receptors, such as SP, VIP, NKA, NKB, NK1R, VIPR1, and VIPR2, were measured by using ELISA kits. The findings indicate that stimulating intestine with Mangxiao or Dahuang can specifically regulate the secretion of SP, VIP, and the receptors in lung tissues. These findings provide a new perspective to interpret the TCM theory of exterior-interior correlation between lung and large intestine.

The theory of exterior-interior correlation between the lung and large intestine was first recorded in *Huang Di Nei Jing* (the Yellow Emperor's Canon of Internal Medicine) and became one of the basic theories of TCM. The theory posits that the specific link between lung and large intestine is meridians. Since the lung meridian of Hand-Taiyin and the large intestine meridian of Hand-Yang ming are a pair of interior-exterior meridians, there is a very close connection between the lung and the large intestine. That is to say, they interact with each other physically and interfere with each other pathologically, such as lung diseases could affect the large intestine [15, 16] and vice versa [17–19]. It has been proved that relaxing intestine by laxative can be very helpful in treating a number of lung diseases including asthma, bronchitis, pneumonia, pleural effusion, pulmonary heart disease, adult respiratory distress syndrome (ARDS), and chronic obstructive pulmonary disease (COPD).

The theory of exterior-interior correlation between the lung and large intestine suggests that they connect with each other directly by meridians, and the connection between the lung meridian and the large intestine meridian is further strengthened by collaterals and branches of the two meridians [20]. The circulation of meridians and meridian points are the physiological basis of acupuncture effects [21]. Some scholars have found that the neural anatomy could be the basis of acupuncture effects and acupuncture stimulation acts on the nerves which could lead to the excretion of substance P, vasoactive intestinal peptide, neuropeptide Y, and so on [22–24]. The finding suggests that peptidergic nerve and neural peptides might play an important role in the exterior-interior correlation between meridians and organs.

Mangxiao and Dahuang, as an osmotic laxative and a stimulant laxative, respectively, have been used in TCM clinical practice for nearly 2000 years. Both of them have an intense effect on contracting the intestinal smooth muscle and increasing peristalsis [25–27], so they are widely prescribed in treating COPD, asthma, and exacerbations or attack other pulmonary diseases. Here, we assumed that herbs like Mangxiao and Dahuang could generate stimuli by stimulating the intestinal tract. Then, enteric nerves receive the stimuli and transmit them to lung through some neural pathways in the body, which ultimately creates a biological effect on regulating lung tissue neuropeptide, such as SP, VIP, and their receptors.

Our findings indicate that by stimulating the large intestine by Mangxiao or Dahuang in rats with COPD, the levels of SP in lung, stomach, and brain tissues and the level of NK1R in lung were reduced. The levels of SP and NK1R in large intestine tissue were increased after stimulating by Dahuang. The levels of VIP in lung and large intestine tissues and VIPR1 and VIPR2 in lung tissue were increased. The level of VIP in brain tissue was reduced. Stimulating the large intestine by Mangxiao in mice with asthma significantly reduced the levels of SP and NK1R in lung tissues but increased the levels of SP and NK1R in large intestine tissues. The levels of VIP in lung, large intestine, and heart tissues and VIPR1 in lung tissues were increased. The levels of NKA and NKB in lung and large intestine tissues of mice with asthma were similar between the control and the model groups.

The results confirmed that after stimulating the large intestine by Mangxiao or Dahuang, SP, NK1R, VIP, VIPR1, and VIPR2 were all significantly increased in large intestine tissue of rats with COPD and mice with asthma. At the same time, the SP and NK1R were markedly decreased, while VIP, VIPR1, and VIPR2 became much higher in lung tissue. Abnormal secretion of SP and VIP could be observed in other organs such as stomach tissues of rats or heart and stomach tissues of mice; however, the receptors did not change obviously, while the NKA and NKB levels were similar in lung tissues of mice with asthma among groups. It indicates that stimulating large intestine with Mangxiao or Dahuang can specifically regulate the secretion of SP, VIP, and the receptors in lung tissues. Our findings provide some new lines of evidence to interpret the theory of exterior-interior correlation between the lung and large intestine.

In conclusion, we have found some new lines of evidence which suggest that a signal conditioning way of *Meridian - nerve - neuropeptide* between lung and large intestine does exist. Because of the complexity of the nervous system and the neural signal transmission, we still need to do further and deeper researches to find out specific nerves and specific ways that associate with the neuropeptide secretion. However, the theory of central neural circuits, proposed by academician Zhang in the research of the mechanism of acupuncture analgesia, may point out a direction for future researches [28].

## Figures and Tables

**Table 1:** SP levels in lung, large intestine, stomach, kidney, spleen, heart, brain, and liver tissues of COPD-like rats (pg/ug, mean ± SD).

Groups	*n*	Lung	Large intestine	Stomach	Kidney	Spleen	Heart	Brain	Liver
Control group	6	0.78 ± 0.07	14.54 ± 3.53	10.51 ± 3.36	102.15 ± 10.67	1.78 ± 0.69	23.50 ± 6.29	2.93 ± 1.64	113.47 ± 9.16
Model group (LPS + cigarette)	7	1.49 ± 0.37*	9.07 ± 2.06*	22.10 ± 4.25*	101.10 ± 24.63	2.35 ± 0.48	15.96 ± 3.25*	7.59 ± 1.60*	130.71 ± 24.02
Mangxiao group (LPS + cigarette + Mangxiao)	6	0.99 ± 0.28^#^	16.93 ± 2.21^#^	6.51 ± 3.78^#^	66.34 ± 7.79^#^	2.09 ± 0.54	16.13 ± 5.42	2.97 ± 1.55^#^	348.79 ± 15.19^#^
Dahuang group (LPS + cigarette + Dahuang)	8	0.78 ± 0.21^#^	15.35 ± 3.35^#^	16.12 ± 7.33^#^	104.48 ± 9.35	2.53 ± 0.23	10.01 ± 3.35^#^	2.76 ± 1.10^#^	144.93 ± 23.53

Means labeled with superscripts were significantly different. *P < 0.05 versus control group, ^#^ *P* < 0.05 versus model group.

**Table 2:** VIP levels in lung, large intestine, stomach, kidney, spleen, heart, brain, and liver tissues of COPD-like rats (pg/ug, mean ± SD).

Groups	*n*	Lung	Large intestine	Stomach	Kidney	Spleen	Heart	Brain	Liver
Control group	6	1.06 ± 0.30	18.99 ± 7.81	19.02 ± 5.26	212.97 ± 29.65	8.90 ± 2.29	34.68 ± 8.01	0.42 ± 0.13	201.58 ± 8.95
Model group (LPS + cigarette)	7	0.51 ± 0.20*	8.24 ± 2.36*	20.42 ± 5.87	209.95 ± 43.53	9.19 ± 1.99	32.08 ± 7.85	0.30 ± 0.12*	220.40 ± 15.72
Mangxiao group (LPS + cigarette + Mangxiao)	6	0.86 ± 0.15^#^	12.58 ± 2.45^#^	10.41 ± 2.94^#^	183.00 ± 35.58	3.09 ± 1.10^#^	21.24 ± 5.59^#^	0.20 ± 0.01^#^	671.90 ± 81.16^#^
Dahuang group (LPS + cigarette + Dahuang)	8	0.96 ± 0.42^#^	12.08 ± 3.29^#^	21.66 ± 7.38	221.89 ± 51.60	8.45 ± 1.28	33.04 ± 9.45	0.21 ± 0.06^#^	245.28 ± 49.94

Means labeled with superscripts were significantly different. *P < 0.05 versus control group, ^#^ *P* < 0.05 versus model group.

**Table 3:** NK1R levels in lung, large intestine, stomach, heart, and brain tissues of COPD-like rats (pg/ug, mean ± SD).

Groups	*n*	Lung	Large intestine	Stomach	Heart	Brain
Control group	6	4.49 ± 0.58	101.33 ± 48.03	116.31 ± 20.12	98.67 ± 22.51	51.24 ± 8.11
Model group (LPS + cigarette)	7	6.43 ± 0.82*	44.86 ± 11.73*	125.80 ± 34.06	106.92 ± 20.47	24.98 ± 14.33*
Mangxiao group (LPS + cigarette + Mangxiao)	6	4.40 ± 1.57^#^	42.05 ± 13.33	40.06 ± 19.45^#^	84.60 ± 20.39	24.97 ± 7.99
Dahuang group (LPS + cigarette + Dahuang)	8	3.29 ± 0.75^#^	73.20 ± 12.10^#^	108.59 ± 31.91	106.70 ± 21.23	20.99 ± 7.11

Means labeled with superscripts were significantly different. *P < 0.05 versus control group, ^#^ *P* < 0.05 versus model group.

**Table 4:** VIPR1 levels in lung, large intestine, stomach, and brain tissues of COPD-like rats (pg/ug, mean ± SD).

Groups	*n*	Lung	Large intestine	Stomach	Brain
Control group	6	3.08 ± 0.45	647.83 ± 86.18	101.83 ± 12.12	52.62 ± 14.00
Model group (LPS + cigarette)	7	1.76 ± 0.43*	253.17 ± 41.75*	64.60 ± 9.77*	45.76 ± 8.31
Mangxiao group (LPS + cigarette + Mangxiao)	6	2.44 ± 0.80	230.44 ± 43.62	66.26 ± 9.02	41.01 ± 11.15
Dahuang group (LPS + cigarette + Dahuang)	8	3.29 ± 1.74^#^	323.27 ± 66.17^#^	67.75 ± 8.76	42.40 ± 11.90

Means labeled with superscripts were significantly different. *P < 0.05 versus control group, ^#^ *P* < 0.05 versus model group.

**Table 5:** VIPR2 levels in lung, large intestine, stomach, and brain tissues of COPD-like rats (pg/ug, mean ± SD).

Groups	*n*	Lung	Large intestine	Stomach	Brain
Control group	6	16.84 ± 3.18	152.97 ± 62.04	132.55 ± 12.57	66.96 ± 3.34
Model group (LPS + cigarette)	7	11.97 ± 2.19*	84.93 ± 22.24*	114.45 ± 31.26	58.10 ± 13.29
Mangxiao group (LPS + cigarette + Mangxiao)	6	19.04 ± 3.91^#^	95.52 ± 14.27	98.16 ± 51.85	53.44 ± 7.89
Dahuang group (LPS + cigarette + Dahuang)	8	16.14 ± 3.17^#^	75.71 ± 8.75	119.63 ± 28.01	67.89 ± 18.69

Means labeled with superscripts were significantly different. *P < 0.05 versus control group, ^#^ *P* < 0.05 versus model group.

**Table 6:** SP levels in lung, large intestine, stomach, kidney, spleen, heart, brain, and liver tissues of asthma mice (pg/mg, *n* = 6, mean ± SD).

Groups	Lung	Large intestine	Stomach	Kidney	Spleen	Heart	Brain	Liver
Control group	5.92 ± 0.58	3.08 ± 0.26	2.98 ± 1.03	1.80 ± 0.54	2.78 ± 0.52	3.47 ± 0.40	2.48 ± 0.60	1.62 ± 0.32
Model group (OVA)	7.27 ± 1.76*	1.74 ± 0.26*	3.10 ± 0.75	1.89 ± 0.44	2.25 ± 0.28	2.13 ± 0.24*	2.08 ± 0.61	0.99 ± 0.13*
Mangxiao group (OVA + Mangxiao)	5.29 ± 2.37^#^	2.49 ± 0.73^#^	4.01 ± 0.99^#^	1.88 ± 0.28	2.38 ± 0.65	2.05 ± 0.72	2.13 ± 0.24	0.78 ± 0.22

Means labeled with superscripts were significantly different. *P < 0.05 versus control group, ^#^ *P* < 0.05 versus model group.

**Table 7:** VIP levels in lung, large intestine, stomach, kidney, spleen, heart, brain, and liver tissues of asthma mice (pg/mg, *n* = 6, mean ± SD).

Groups	Lung	Large intestine	Stomach	Kidney	Spleen	Heart	Brain	Liver
Control group	5.84 ± 0.35	2.10 ± 0.36	3.22 ± 0.35	1.53 ± 0.27	1.90 ± 0.27	2.74 ± 0.84	2.10 ± 0.31	1.23 ± 0.17
Model group (OVA)	4.04 ± 0.26*	1.43 ± 0.28*	3.80 ± 0.11*	1.50 ± 0.18	2.15 ± 0.30	1.27 ± 0.35*	1.53 ± 0.30*	0.96 ± 0.15
Mangxiao group (OVA + Mangxiao)	5.09 ± 0.60^#^	1.95 ± 0.35^#^	3.26 ± 0.50^#^	1.76 ± 0.20	1.96 ± 0.25	2.16 ± 0.54^#^	1.56 ± 0.38	1.16 ± 0.39

Means labeled with superscripts were significantly different. *P < 0.05 versus control group, ^#^ *P* < 0.05 versus model group.

**Table 8:** NK1R levels in lung, large intestine, stomach, and heart tissues of OVA allergic asthma mice (pg/ug, *n* = 6, mean ± SD).

Groups	Lung	Large intestine	Stomach	Heart
Control group	0.96 ± 0.19	1.16 ± 0.23	6.80 ± 2.36	29.11 ± 5.28
Model group (OVA)	1.18 ± 0.19*	1.38 ± 0.32	9.40 ± 1.13*	35.38 ± 10.05
Mangxiao group (OVA + Mangxiao)	0.97 ± 0.13^#^	1.86 ± 0.27^#^	10.84 ± 1.38	35.60 ± 13.06

All values are expressed as mean ± SD. Means labeled with superscripts were significantly different. *P < 0.05 versus control group, ^#^ *P* <0.05 versus model group.

**Table 9:** VIPR1 levels in lung, large intestine, stomach, and heart tissues of asthma mice (pg/ug, *n* = 6, mean ± SD).

Groups	Lung	Large intestine	Stomach	Heart
Control group	5.72 ± 0.62	1.06 ± 0.13	2.67 ± 0.85	7.59 ± 3.89
Model group (OVA)	3.93 ± 0.47*	0.73 ± 0.15*	3.15 ± 0.68	7.26 ± 3.10
Mangxiao group (OVA + Mangxiao)	5.26 ± 0.65^#^	0.96 ± 0.11^#^	10.089 ± 4.78^#^	8.46 ± 0.54

Means labeled with superscripts were significantly different. *P < 0.05 versus control group, ^#^ *P* < 0.05 versus model group.

**Table 10:** VIPR2 levels in lung, large intestine, stomach, and heart of asthma mice (pg/ug, *n* = 6, mean ± SD).

Groups	Lung	Large intestine	Stomach	Heart
Control group	1.47 ± 0.19	1.18 ± 0.17	2.84 ± 1.09	16.59 ± 8.78
Model group (OVA)	1.11 ± 0.41*	1.22 ± 0.48	6.00 ± 1.30*	24.32 ± 13.55
Mangxiao group (OVA + Mangxiao)	1.43 ± 0.15	1.32 ± 0.19	5.85 ± 3.04	29.57 ± 17.12

Means labeled with superscripts were significantly different. *P < 0.05 versus control group, ^#^ *P* < 0.05 versus model group.

**Table 11:** NKA levels in lung, large intestine, stomach, kidney, spleen, heart, brain, and liver tissues of asthma mice (pg/mg, *n* = 6, mean ± SD).

Groups	Lung	Large intestine	Stomach	Kidney	Spleen	Heart	Brain	Liver
Control group	0.88 ± 0.23	0.27 ± 0.06	0.39 ± 0.08	0.24 ± 0.03	0.30 ± 0.05	0.41 ± 0.05	0.26 ± 0.06	0.17 ± 0.03
Model group (OVA)	0.87 ± 0.07	0.28 ± 0.06	0.37 ± 0.06	0.21 ± 0.05	0.27 ± 0.06	0.30 ± 0.03*	0.25 ± 0.03	0.14 ± 0.01*
Mangxiao group (OVA + Mangxiao)	0.75 ± 0.09	0.19 ± 0.05^#^	0.30 ± 0.02	0.24 ± 0.04	0.30 ± 0.04	0.29 ± 0.05	0.25 ± 0.05	0.15 ± 0.03

Means labeled with superscripts were significantly different. *P < 0.05 versus control group, ^#^ *P* < 0.05 versus model group.

**Table 12:** NKB levels in lung, large intestine, stomach, kidney, spleen, heart, brain, and liver tissues of asthma mice (pg/mg, *n* = 6, mean ± SD).

Groups	Lung	Large intestine	Stomach	Kidney	Spleen	Heart	Brain	Liver
Control group	2.97 ± 0.36	1.16 ± 0.35	1.48 ± 0.08	0.80 ± 0.24	1.02 ± 0.22	0.64 ± 0.26	1.02 ± 0.14	0.27 ± 0.09
Model group (OVA)	2.81 ± 0.64	1.33 ± 0.43	1.55 ± 0.34	0.68 ± 0.15	0.87 ± 0.22	0.43 ± 0.24	0.72 ± 0.13*	0.23 ± 0.06
Mangxiao group (OVA + Mangxiao)	2.07 ± 0.45^#^	0.22 ± 0.08^#^	0.62 ± 0.21^#^	0.76 ± 0.12	0.55 ± 0.20^#^	0.75 ± 0.24	0.84 ± 0.16	0.27 ± 0.06

Means labeled with superscripts were significantly different. *P < 0.05 versus control group, ^#^ *P* < 0.05 versus model group.
